# Supplementary material for: Dipolar order mediated 1H → 13C cross-polarization for dissolution-dynamic nuclear polarization
Source: Magn Reson (Gott). 2020 May 20;1(1):89–96. doi: 10.5194/mr-1-89-2020 (PMC10500725; doi:10.5194/mr-1-89-2020)
Supplement: The supplement related to this article is available online at: https://doi.org/10.5194/mr-1-89-2020-supplement. [file mr-1-89-supplement.pdf]

Supplement of Magn. Reson., 1, 89–96, 2020
https://doi.org/10.5194/mr-1-89-2020-supplement
© Author(s) 2020. This work is distributed under
the Creative Commons Attribution 4.0 License.

Supplement of

Dipolar order mediated $^{1}\mathrm{H} \rightarrow {}^{13}\mathrm{C}$ cross-polarization for dissolution-dynamic nuclear polarization

Stuart J. Elliott et al.

Correspondence to: Stuart J. Elliott (stuart-james.elliott@univ-lyon1.fr)

The copyright of individual parts of the supplement might differ from the CC BY 4.0 License.

Contents

1 Cross-Polarization 3

2 B_1-field Optimizations 4

3 References 5

1. Cross-Polarization

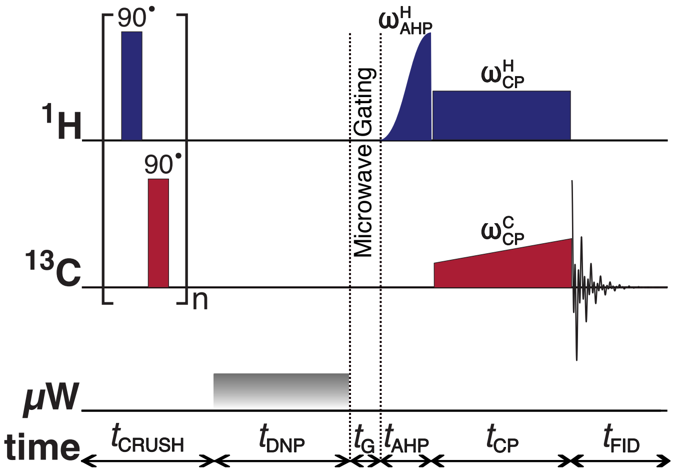

Figure S1: Schematic representation of the cross-polarization (CP) *rf*-pulse sequence adapted to *d*DNP experiments used for transferring ^{1}H polarization to ^{13}C heteronuclei. The experiments used the following parameters, chosen to maximize ^{1}H-^{13}C population conversion: n = 250; t_{DNP} = 640 s; t_{G} = 0.5 s; $\omega_{AHP}^{H}/2\pi$ = 27.8 kHz; t_{AHP} = 175 μs; $\omega_{CP}^{H}/2\pi$ = 18.3 kHz; $\omega_{CP}^{C}/2\pi$ = 21.4 kHz; t_{CP} = 7 ms. AHP = adiabatic half-passage [1]. The $\pi/2$ crusher *rf*-pulses use a thirteen-step phase cycle to remove residual magnetization at the beginning of each experiment: $\{0, \pi/18, 5\pi/18, \pi/2, 4\pi/9, 5\pi/18, 8\pi/9, \pi, 10\pi/9, 13\pi/9, \pi/18, 5\pi/3, 35\pi/18\}$. The resonance offset was placed at the centre of the ^{1}H and ^{13}C NMR peaks.

Figure S1 shows the typical cross-polarization (CP) *rf*-pulse sequence configured for use in *d*DNP experiments to transfer polarization from ^{1}H spins to insensitive ^{13}C heteronuclei [2]. As detailed in the main text, the CP *rf*-pulse sequence achieved a ^{13}C polarization of P(^{13}C) ≃ 20.4% after 640 s of direct ^{1}H DNP at 1.2 K prior to a single step transfer of polarization to the ^{13}C spin bath.

2. B_1-field Optimization

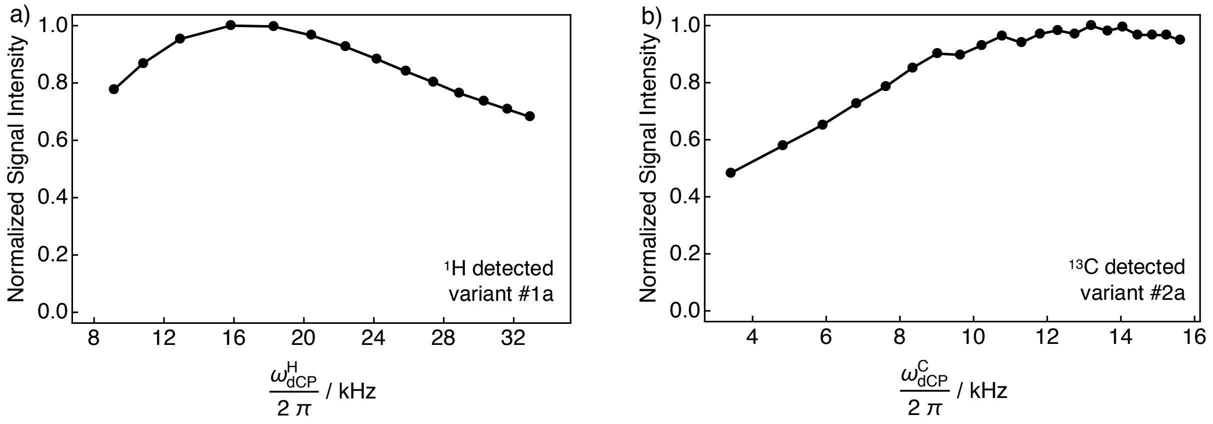

Figure S2: Experimental NMR signal intensities of **I** as a function of the (a) ^{1}H *d*CP *rf*-pulse amplitude $\omega_{\mathrm{dCP}}^{\mathrm{H}}$ and (b) ^{13}C *d*CP *rf*-pulse amplitude $\omega_{\mathrm{dCP}}^{\mathrm{C}}$ acquired at 7.05 T (^{1}H nuclear Larmor frequency = 300.13 MHz, ^{13}C nuclear Larmor frequency = 75.47 MHz) and 1.2 K. The experiments in (a) were acquired with two transients per data point, whilst the experiments in (b) were acquired with a single transient per data point. All data points were normalized with respect to the highest intensity NMR signal.

Figure S2a displays the ^{1}H monitored optimization for the build-up of ^{1}H-^{1}H dipolar order performed by using *variant #1a* of the *d*CP *rf*-pulse sequence demonstrated in Figure 2a of the main text. The experimental integrals are plotted against the ^{1}H *rf*-pulse amplitude $\omega_{\mathrm{dCP}}^{\mathrm{H}}$ and were acquired on the ^{1}H *rf*-channel with $t_{\mathrm{dCP}}^{\mathrm{H}}$ = 25 μs (black circles). The ^{1}H NMR signal increases until a maximum NMR signal intensity, which corresponds to the optimal generation of ^{1}H-^{1}H dipolar order, is achieved at $\omega_{\mathrm{dCP}}^{\mathrm{H}} \simeq$ 16.4 kHz, after which the NMR signal intensity decreases at higher *rf*-pulse nutation frequencies.

Figure S2b exhibits the ^{13}C observed optimization for the creation of ^{13}C magnetization realized by implementing *variant #2a* of the *d*CP *rf*-pulse sequence detailed in Figure 3a of the main text. The experimental integrals are plotted against the ^{13}C *rf*-pulse amplitude $\omega_{\mathrm{dCP}}^{\mathrm{C}}$ and were obtained on the ^{13}C *rf*-channel with $\omega_{\mathrm{dCP}}^{\mathrm{H}} \simeq$ 16.4 kHz, $t_{\mathrm{dCP}}^{\mathrm{H}}$ = 25 μs and $t_{\mathrm{dCP}}^{\mathrm{C}}$ = 39 ms (black circles). The ^{13}C NMR signal builds until a maximum NMR signal intensity, which corresponds to the optimal creation of ^{13}C magnetization, is reached at $\omega_{\mathrm{dCP}}^{\mathrm{C}} \simeq$ 13.2 kHz, after which the NMR signal intensity plateaus at increased *rf*-pulse nutation frequencies.

3. References

[1] A. Bornet, A. Pinon, A. Jhajharia, M. Baudin, X. Ji, L. Emsley, G. Bodenhausen, J. H. Ardenkjær-Larsen and S. Jannin, *Phys. Chem. Chem. Phys.*, **2016**, 18, 30530-30535.

[2] A. Pines, M. Gibby and J. Waugh, *Chem. Phys. Lett.*, **1972**, 15, 373-376.
